# Observance of radiation protection principles in Iranian dental schools

**Published:** 2010

**Authors:** Amir Eskandarlou, Karim Ghazi-khanlou Sani, Nima Rostampour

**Affiliations:** a Assistant Professor of Dental and Maxillofacial Radiology, School of Dentistry, Department of Radiology, Hamadan University of Medical Sciences, Hamadan, Iran; b MS in Medical Physics, School of Para-medicine, Department of Radiology, Hamadan University of Medical Sciences, Hamadan, Iran; c MS in Medical Physics, School of Medicine, Department of Medical Physics, Hamadan University of Medical Sciences, Hamadan, Iran

The use of radiation in the medical practice has evolved since its beginning and 30% to 50% of medical decisions are affected by radiologic examinations.^1^ However, the hazards of ionizing radiation are irrefutable.^1^ Dental radiography represents one of the most frequently used radiologic examinations in the industrialized world.^2^ This type of radiography stands for 25% of the radiologic examinations performed in the European Union.^3^ It means that the dose to the population as a whole is considerable. Therefore, some particular attention should be paid to radiation safety.^2^,^3^

The European Commission (EC) and National Radiation Protection Board (NRPB) have been collected some guidelines about radiation protection in dentistry.^4^,^5^ The aim of this study was to investigate the observance of radiation protection principles in Iranian dental schools.

This cross-sectional questionnaire-based study was conducted between September 2008 and February 2009. A questionnaire was conducted regarding EC and NRPB recommendations about radiation protection in dental radiography. The radiology departments of all dental school of Iran (18 schools) surveyed in this study.

Agreement of the results with some recommendations of EC and NRPB are shown in table 1.

**Table 1:** Agreements of dental schools status with guidelines of EC and NRPB in different aspects

	Agreement N (%)		
Item	Intra-oral radiography	Extra-oral radiography
1- Utilizing the rectangular collimator	2 (11.1)	--------
2- PID length	17 (94.4)	--------
3- Existence of protective covers	18 (100)	18 (100)
4- Policy for use of protective covers	5 (27.8)	4 (22.2)
5- Responsible person for radiography	12 (66.7)	16 (88.9)
6- Existence of radiation monitoring systems	17 (94.4)	17 (94.4)
7- Responsible person for holding disable patients	18 (100)	18 (100)
8- Regular measurements of films sensitivity	5 (27.8)	1 (5.6)
9- Measurements of darkroom lighting and safelight condition	3 (16.7)	2 (11.1)
10- Personnel dose received monitoring	17 (94.4)	18 (100)
11- Quality control and output measurements of x-ray sets	0 (0)	2 (11.1)
12- Responsible person about quality control programs	13 (72.2)	13 (72.2)
13- Quality control periods for base density of films	1 (5.6)	5 (27.8)
14- Quality control periods for darkroom lighting	3 (16.7)	3 (16.7)
15- Output measurements of x-ray sets	2 (11.1)	2 (11.1)

The findings about radiation protection principles observance in dental radiography point to a slightly better situation than those mentioned by former similar studies in Iran.

The current survey emphasizes on the need for further consideration of radiation protection principles, especially on the field of quality control and quality assurance programs, in dental schools of Iran. If quality control and quality assurance problems are corrected, the situation can be improved.
